# Impact of Maternal Ketogenic Diet on NLRP3 Inflammasome Response in the Offspring Brain

**DOI:** 10.3390/nu15081994

**Published:** 2023-04-21

**Authors:** Sevsen Altınöz, Serap Cilaker Micili, Sıla Soy, Defne Engür, Bora Baysal, Abdullah Kumral

**Affiliations:** 1 Department of Pediatrics, Faculty of Medicine, Dokuz Eylul University, Izmir 35330, Turkey; 2 Department of Histology and Embryology, Faculty of Medicine, Dokuz Eylul University, Izmir 35330, Turkey; 3 İzmir International Biomedicine and Genome Center, Dokuz Eylul University, Izmir 35330, Turkey; 4 Division of Neonatology, Department of Pediatrics, Izmir Faculty of Medicine, University of Health Sciences, Izmir 35330, Turkey; 5 Division of Neonatology, Department of Pediatrics, Faculty of Medicine, Istinye University, Istanbul 34517, Turkey; 6 Division of Neonatology, Department of Pediatrics, Faculty of Medicine, Dokuz Eylul University, Izmir 35330, Turkey

**Keywords:** ketogenic diet, pregnancy, lactation, neonate, inflammasome, sepsis, neuroinflammation, nucleotide-binding domain, leucine-rich-containing family, pyrin domain-containing-3 (NLRP3) inflammasome, microglia, brain

## Abstract

The effects of maternal diet on the neuroimmune responses of the offspring remain to be elucidated. We investigated the impact of maternal ketogenic diet (KD) on the NLRP3 inflammasome response in the offspring’s brain. C57BL/6 female mice were randomly allocated into standard diet (SD) and ketogenic diet (KD) groups for 30 days. After mating, the presence of sperm in the vaginal smear was considered day 0 of pregnancy, and female mice continued their respective diets during pregnancy and the lactation period. Following birth, pups were further allocated into two groups and given either LPS or intraperitoneal saline on postnatal (PN) days 4, 5 and 6; they were sacrificed on PN11 or PN21. Neuronal densities were significantly lower globally in the KD group when compared to the SD group at PN11. Neuronal density in the prefrontal cortex (PFC) and dentate gyrus (DG) regions were also significantly lower in the KD group when compared to the SD group at PN21. Following administration of LPS, the decrease in the neuronal count was more prominent in the SD group when compared to the KD group in the PFC and DG regions at PN11 and PN21. NLRP3 and IL-1β were higher in the KD group than in the SD group at PN21 in the PFC, CA1 and DG regions, and were significantly lower in the DG region of the KD group especially when compared to the SD group following LPS. Results of our study reveal that maternal KD negatively affects the offspring’s brain in the mouse model. The effects of KD exhibited regional variations. On the other hand, in the presence of KD exposure, NLRP3 expression after LPS injection was lower in the DG and CA1 areas but not in the PFC when compared to SD group. Further experimental and clinical studies are warranted to elucidate the molecular mechanisms underlying the impact of antenatal KD exposure and regional discrepancies on the developing brain.

## 1. Introduction

The implications of maternal nutrition on fetal brain development are well established [1]. However, the impact of maternal dietary composition on the neuroimmune response of offspring has not been as well-characterized [2]. Although there are studies focusing on the effects of maternal macronutrient and micronutrient intake in pregnancy on fetal brain development and offspring behavior [1,3], a considerable knowledge gap still remains regarding the impact of maternal dietary composition on cellular responses in offspring brains.

Ketogenic diet (KD), i.e., a high-fat, low-carb, appropriate protein diet, has traditionally been employed largely as a therapeutic option for children with intractable convulsions [4]. Adult patients with epilepsy have recently been adopting KD due to its efficacy and considerable advantages over anti-epileptic medicines [5]. The KD has also gained popularity as a lifestyle diet for maintaining weight and supporting muscle mass in healthy individuals and is used in the treatment of obesity [6]. Due to rising and continuous use of the KD by adults, particularly women, concerns have been raised about its effects on offspring [7].

Microglia are the resident immune cells of the brain, which constantly seek signals from the external environment and respond to nutritional immunomodulators [8]. Microglial cells can adopt different phenotypes and exert diverse responses in the central nervous system, which can be modulated by dietary components as well [9].

The nucleotide-binding domain, leucine-rich-containing family, pyrin domain-containing 3 (NLRP3) inflammasome is a multiprotein complex that can be triggered by various stimuli and drives inflammatory responses in the central nervous system [10]. Recent studies indicate that the NLRP3 inflammasome response in microglial cells can be modulated through substrate availability and brain energy metabolism in adults [11]. Ketone bodies are known to inhibit microglial NLRP3 inflammasome activation in rodent models of Alzheimer’s disease [12]; however, the effect of maternal KD on offspring responses to inflammation in the brain is not established. In the present study, we aimed to investigate the impact of KD during pregnancy and the lactation period on NLRP3 inflammasome response in the offspring brain following LPS administration.

## 2. Materials and Methods

### 2.1. Animals and Study Design

This study was conducted in accordance with the guidelines provided by the Experimental Animal Laboratory and approved by the Animal Care and Use Committee of the Dokuz Eylul University School of Medicine (Protocol number: 21/2021) and the ARRIVE guidelines 10.0 [13]. Animals were housed in a temperature- and humidity-controlled animal facility with a 12-h dark and light cycle. All pregnant females and their offspring had free access to food and water throughout the experiments.

**Figure 1:**
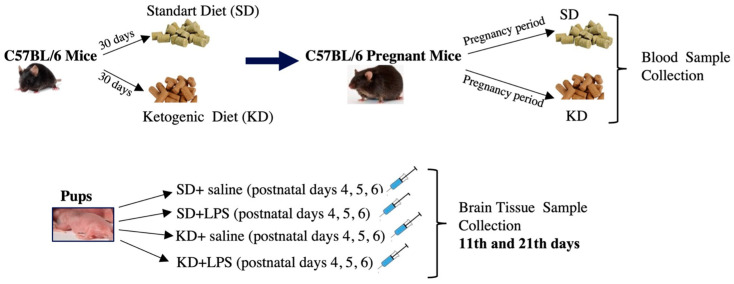
Schematic illustration of the experimental design of the study.

Six-week-old C57BL/6 type female mice were randomly allocated into the standard diet (SD) and ketogenic diet (KD) groups. Dams were fed by their respective diets for 30 days and then naturally mated by single males. The presence of sperm in the vaginal smear was considered day 0 of pregnancy, and female mice continued their respective diets during pregnancy and the lactation period. Maternal metabolic status was observed through body weight, blood glucose, ketone, cholesterol and triglyceride concentrations via the collection of blood from the tail vein. Maternal blood glucose and β ketones (β-hydroxybutyrate) concentrations were measured using an On Call GK Dual ketone and glucose meter, which requires 0.6–1.5 µL of whole blood taken from the tail vein per test. Maternal serum total cholesterol and triglyceride levels were measured at the day of sacrifice using approximately 0.5 cc of whole blood taken via cardiac puncture. Following birth, pups whose mothers were fed a SD and KD were divided into two groups after birth, and four groups were formed: SD, SL + LPS, KD and KD + LPS (n = 14). A total of 6 mg/kg intraperitoneal injection (ip) LPS (L2880, Sigma Aldrich) or ip saline for three consecutive days was given on postnatal (PN) days 4, 5 and 6. Half of these groups (7 animals from each group) were sacrificed on the 11th day and the other half on the 21st day. Figure 1 illustrates the experimental design of the study. Pups were monitored for daily weight gain.

### 2.2. Diet

The SD and KD were both purchased from Research Diets Inc., New Brunswick, NJ, USA (www.researchdiets.com, accessed on 1 March 2023). The SD consisted of 5% fat, 75% carbohydrate and 20% protein, providing 3.1 Kcal/gr. The KD consisted of 67.2% fat, 0.2% carbohydrate and 17.6% protein, which is equivalent to a 3:1 ratio—weight of fat to combined protein and carbohydrate— and provides 6.7 Kcal/gr (very low carbohydrate ketogenic rodent diet, product number: D12369B). All mice were allowed unlimited access to their food and water prior to mating and during gestation and the lactation period [14].

### 2.3. Histomorphological Evaluation

Pups were sacrificed, at one of two time-points—PN11, i.e., corresponding to the end of rapid brain growth, or PN21, i.e., soon after the onset of the rapid myelination period. Brains were extracted, weighed and fixed in 10% formaline. Routine histological methods were applied to tissues embedded in paraffin blocks. Coronal sections of 5 µm were obtained using a rotary microtome (Leica RM2135, Berlin, Germany). Sections were obtained from plates 8–9 and 20–22, corresponding to the prefrontal cortex (PFC) and hippocampus (CA1, dentate gyrus (DG) for neuronal density and immunohistochemistry), respectively, and in accordance with the rat atlas of Paxinos and Watson [15].

### 2.4. Neuronal Density Measurement 

For the estimation of neuronal densities, images were analyzed by using a computer assisted Image-J analyze program. The counting frame was randomly placed on the image analyzer system monitor three times; the number of neurons stained by cresyl violet were counted in a square area of ~1.875 μm^2^ [16]. 

### 2.5. Immunohistochemistry

Following deparaffinization and rehydration, sections were treated with a 10-mM citrate buffer (Cat No.AP-9003-125 Labvision). Endogenous peroxidase was inactivated in 3% H_2_O_2_, then incubated with normal serum blocking solution. Sections were incubated over 18 h at +4 °C overnight with AIF-1(allograft inflammatory factor 1, AB-70353, Elabscience, Texas, USA), NLRP3 (AG-20B-0014-C100, Adipogen, San Diego, CA, USA), and IL1β (SC7884, Santa Cruz, Darmstadt, Germany) primer antibodies. After washing, sections were then incubated with biotinylated IgG and then with streptavidin conjugated to horseradish peroxidase (Invitrogen 85–9043). Sections were stained with DAB and counter-stained with Mayer’s hematoxylin and analyzed using a light microscope [17].

### 2.6. Quantification of Immunohistochemical Data

Immunostaining intensity was categorized into the following scores: 0 (no staining), 1 (weak, but detectable staining), 2 (moderate staining) and 3 (intense staining). The H-score value was derived for each specimen by calculating the sum of the percentage of cells. It was categorized by the intensity of staining and multiplied by its respective score by means of the formula: H-score = ∑Pi (i + 1), where i is the intensity of staining with a value of 1, 2 or 3 (weak, moderate or strong, respectively) and Pi is the percentage of stained cells for each intensity, varying from 0% to 100%. For each slide, five different fields were evaluated microscopically at a 200× magnification. H-score evaluation was performed by at least two investigators independently who were blinded to the source of the samples as well as to each other’s results, and the average score was determined [16].

### 2.7. Statistical Analysis

Data were analyzed using SPSS version 25.0 (IBM, Armonk, NY, USA). Normality of the distribution was evaluated with the Kolmogorov–Smirnov test. Values are presented as the means ± medians and 25th–75th quartiles (25–75%). For the comparison of groups of more than two, the Kruskal–Wallis H Test was used with the Mann–Whitney U post hoc test to determine the group that caused the difference. *p* < 0.05 was considered significant.

## 3. Results

### 3.1. Maternal Weight Gain

Maternal weight gain at gestational days (GDs) 0, 10 and 20 was similar in both the KD and SD groups, while it was significantly lower in the KD group when compared to the SD group during postpartum (PP) days 10 and 20 (Table 1). 

### 3.2. Maternal Blood Biochemistry

Blood glucose and ketone levels were measured at GDs 2, 10 and 18; total blood cholesterol and triglyceride levels were measured at the end of the lactation period (PP20). Blood β-hydroxybutyrate (BHB) levels were significantly higher in the KD group when compared to the SD group on all three occasions (*p* < 0.05) (Table 2). Blood glucose levels were similar in both groups during the first half of the pregnancy, whereas they were lower in the KD group when compared to the SD group in the second half (Table 3) (*p* < 0.05). The lowest measured blood glucose level was 81 mg/dl, meaning hypoglycemia was not observed in any group. Blood total cholesterol and triglyceride levels were measured once at GD20 and were significantly higher in the KD group when compared to the SD group (71.8 ± 15.3 and 109.6 ± 50.4 vs. 121.3 ± 19.7 and 59.8 ± 9.04, respectively) (*p* < 0.05) (Table 4). 

### 3.3. Litter Numbers, Body and Brain Weights in the Offspring

The litter numbers were comparable, and the body weights of the pups were similar at PN0 and PN2. Although body weights on days PN10 and PN20 were also similar among the KD and SD groups, weight gain was lower in the SD group when compared to the KD group in the presence of LPS insult (*p* < 0.05). (Table 5)

When brain weights at PN11 were compared, the lowest brain weight was found in the SD + LPS group, and the brain weight of the pups in the KD + LPS group were significantly higher than those in the SD + LPS group (*p* < 0.05). Additionally, at PN21, KD pups displayed higher brain weights when compared to SD pups, (*p* < 0.05), and the brain weight of the pups in the KD + LPS group were not lower when compared to the SD + LPS group (Table 6).

### 3.4. Neuronal Density Results

**Figure 2:**
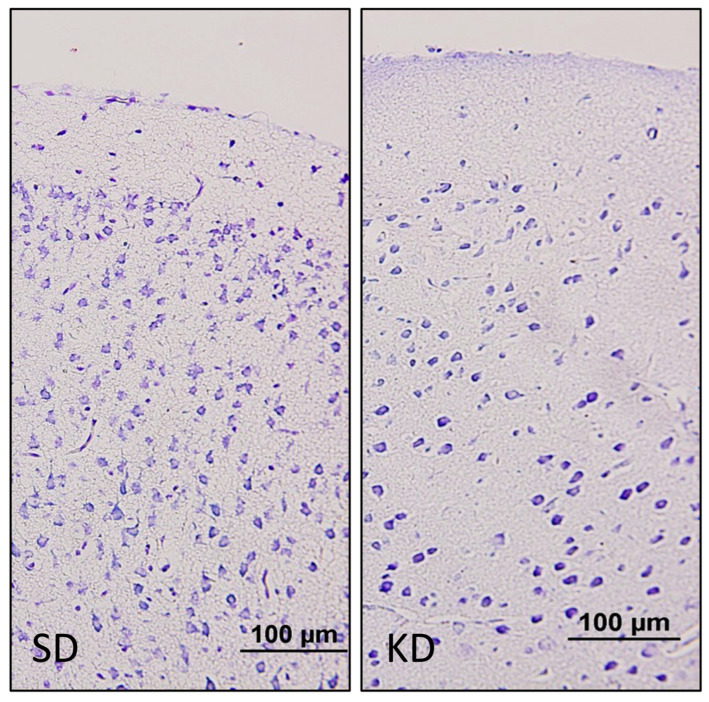
Cresyl violet staining of the prefrontal cortex. The neuronal density was lower in the KD group when compared to the SD group at PN11. (20×).

**Figure 3:**
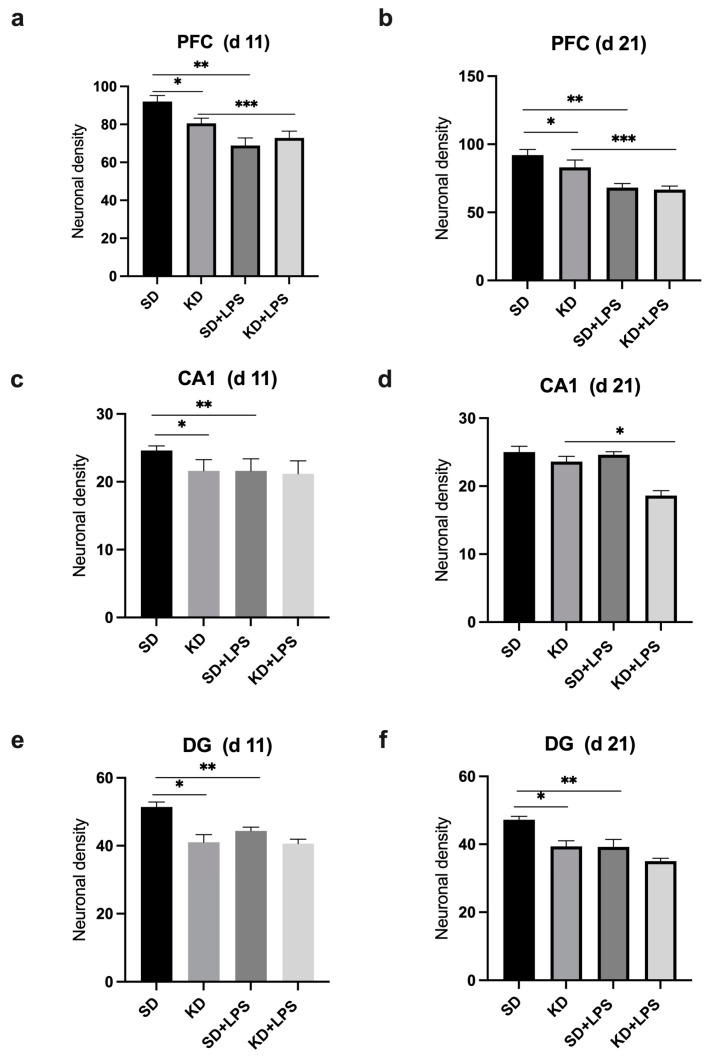
Comparison of neuronal densities in the prefrontal cortex (PFC), CA1 and dentate gyrus (DG) regions at postnatal days PN11 and PN21. *, **, *** *p* < 0.05. Neuronal density in the PFC (**a**), CA1 (**c**) and DG (**e**) regions were found to be lower in the KD group when compared to the SD group at PN11 and neuronal density in the PFC (**b**), CA1(**d**) and DG (**f**) regions were found to be lower in the KD group at PN21.

Neuronal density in the PFC, CA1 and DG regions were found to be significantly lower in the KD group when compared to the SD group at PN11 (*p* < 0.05) (Figure 2 and Figure 3a,c,e). Neuronal density in the PFC and DG regions was also found to be significantly lower in the KD group when compared to the SD group at PN21, (*p* < 0.05) (Figure 3b,f). However, it was observed that neuronal density decreased in all regions with LPS application in the SD + LPS and KD + LPS groups compared to the SD and KD groups. A significant difference was observed between the SD and SD + LPS groups in the PFC and DG regions (Figure 3b,f) and between the KD and KD + LPS groups (*p* < 0.05) (Figure 3d) in the PFC and CA regions (*p* < 0.05) (Figure 3b,d).

**Figure 4:**
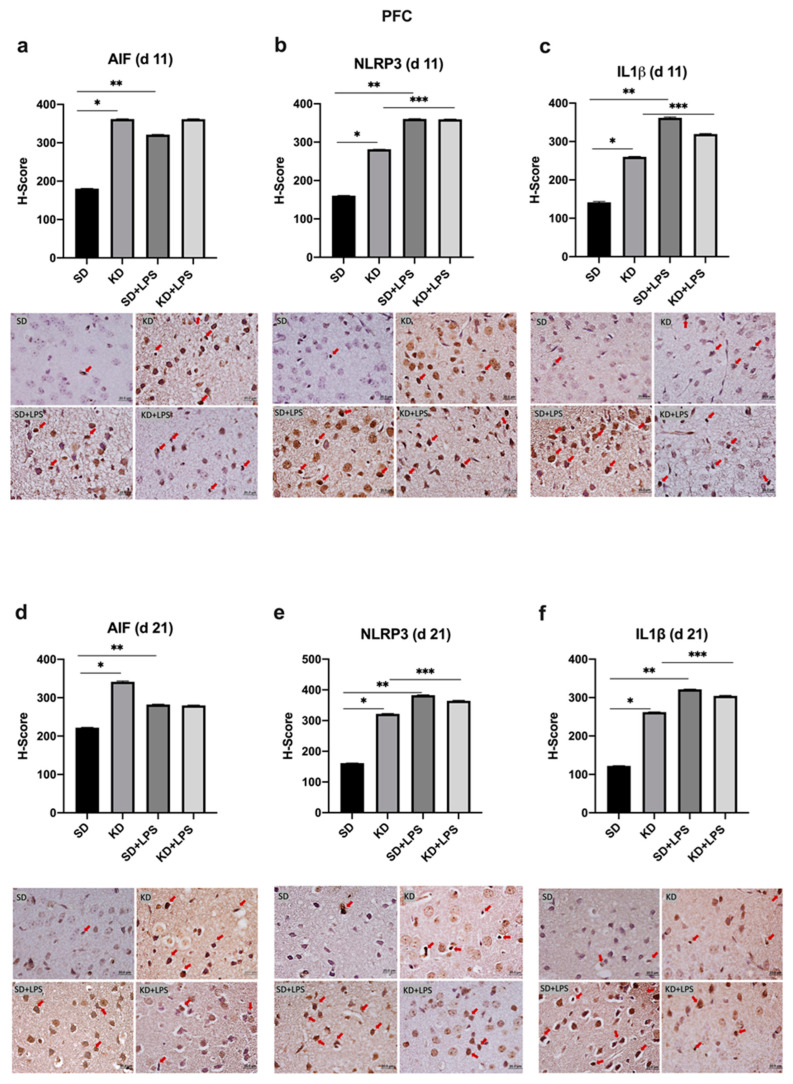
Immunohistochemical H-scoring and staining of the prefrontal cortex (PFC) with AIF, NLRP3 and IL1β on postnatal days PN11 and PN21. IHC images are compatible with the graphic above. Red arrows indicate immunopositive cells. *, **, *** *p* < 0.05. AIF1, NLRP3 and IL1β expression was increased in the PFC of the KD group when compared to the SD group at PN11 and PN21 (**a**–**f**). NLRP3 and IL1β expression was increased in the LPS-treated groups when compared to saline treated groups (**b**,**c**,**e**,**f**).

**Figure 5:**
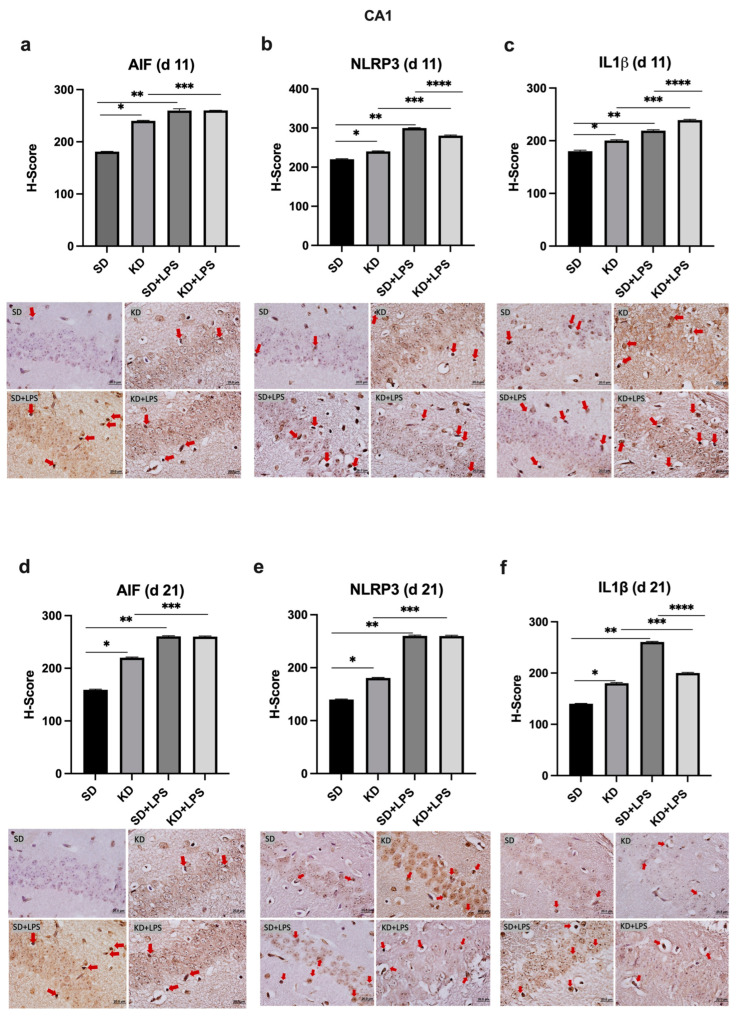
Immunohistochemical H-scoring and staining of the CA1 region of the hippocampus with AIF, NLRP3 and IL1β on postnatal days PN11 and PN21. IHC images are compatible with the graphic above. Red arrows indicate immunopositive cells. *, **, ***,**** *p* < 0.05. AIF1, NLRP3 and IL1β expression was increased in the CA1 of the KD group when compared to the SD group at PN11 and PN21 (**a**–**f**). AIF, NLRP3 and IL1β expression was increased in the LPS-treated groups when compared to saline treated groups (**a**–**f**).

**Figure 6:**
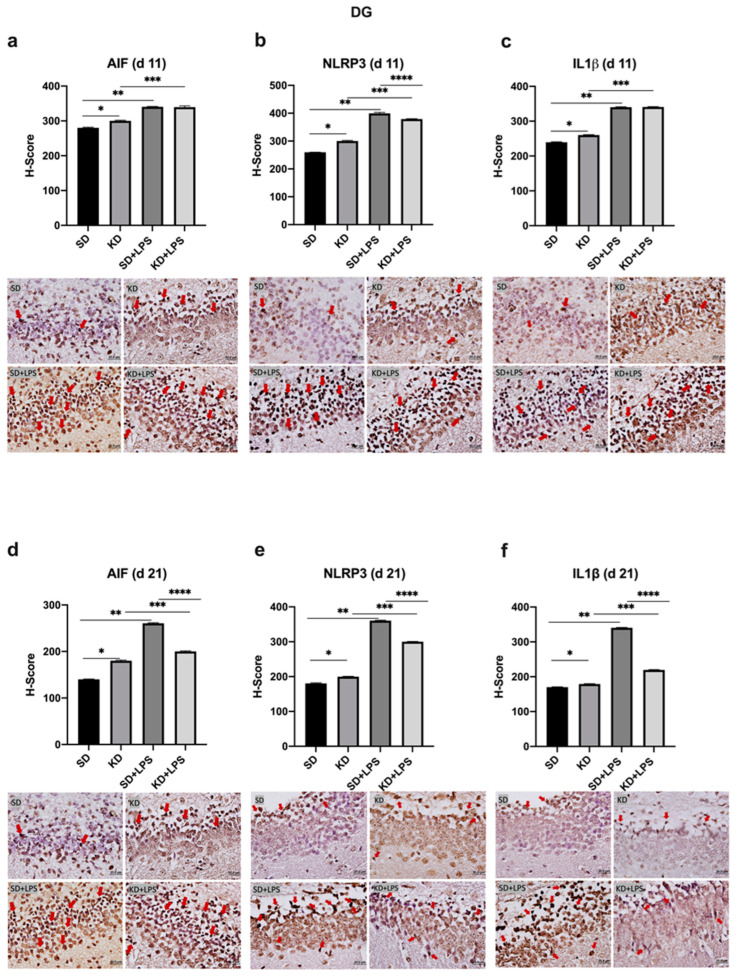
Immunohistochemical H-scoring and staining of the dentate gyrus (DG) with AIF, NLRP3 and IL1β on postnatal days PN11 and PN21. IHC images are compatible with the graphic above. Red arrows indicate immunopositive cells. *, **, ***,**** *p* < 0.05. AIF1, NLRP3 and IL1β expression was increased in the DG of the KD group when compared to the SD group at PN11 and PN21 (**a**–**f**). AIF, NLRP3 and IL1β expression was increased in the LPS-treated groups when compared to saline treated groups (**a**–**f**). NLRP3 and IL1β expression was decreased in KD + LPS group when compared to SD + LPS group at PN21 (**e**,**f**).

At PN11, microglial marker AIF1 expression was increased in the PFC, CA1 and DG regions of the KD group when compared to the SD group; however, there was no significant difference between the KD + LPS and SD + LPS groups (Figure 4a, Figure 5a and Figure 6a).

NLRP3 and IL-1β expression were higher in the KD group when compared to the SD group and also higher in the LPS-treated groups when compared to the saline treated groups at PN11 in the PFC, CA1 and DG regions (*p* < 0.05) (Figure 4b,c, Figure 5b,c and Figure 6b,c). However, NLRP3 expression was significantly lower in the KD + LPS group when compared to the SD + LPS group in the CA1 and DG regions (*p* < 0.05), and no significant difference was found among the KD + LPS and SD + LPS groups in the PFC region at PN11 (Figure 4b, Figure 5b and Figure 6b). IL-1β expression was higher in the KD + LPS group when compared to the SD + LPS group in the CA1 region (*p* < 0.05), and no significant difference was found among the KD + LPS and SD + LPS groups in the PFC and DG regions at PN11 (Figure 4c, Figure 5c and Figure 6c).

At PN21, AIF1 expression was still higher in the PFC, CA1 and DG regions of the KD group when compared to the SD group; however, no significant difference was demonstrated among the KD + LPS and SD + LPS groups in the PFC and CA1 regions (Figure 4a, Figure 5a and Figure 6a), and AIF1 expression was even lower in the KD + LPS group when compared to the SD + LPS group in the DG region (*p* < 0.05) (Figure 4d, Figure 5d and Figure 6d).

At PN21, NLRP3 and IL-1β expressions in the DG region were also lower in the KD + LPS group when compared to the SD + LPS group (*p* < 0.05) (Figure 5f and Figure 6e). NLRP3 and IL-1β expressions were comparable among the KD + LPS and SD + LPS groups in the PFC region (Figure 3f and Figure 4e).

## 4. Discussion

It is widely known that maternal diet has an impact on the immunological development of the fetus. This effect may result from changing the metabolic status or altering the levels of circulating immune active molecules. Some dietary components can also interact with certain transcription factors that regulate the inflammation process and modulate fetal inflammatory responses [18,19]. The KD is becoming more popular, especially among women who are of a childbearing age. However, its safety during pregnancy and lactation is largely unknown. There are few studies in the literature that examine how a gestational KD affects neonatal brains [7,20,21]. Early studies on prenatal ketosis tended to focus on the etiology, such as maternal starvation, extended fasting or diabetes. However, they differ from the consistent ketosis that follows a KD which supplies adequate calories and minerals [20]. Later studies on antenatal KD exposure mainly focused on the structural changes in the offspring’s brain [7,22] and behavioral outcome in adulthood [21,23]. In the present study, we investigated the effects of antenatal KD on brain histology and NLRP3 response in the presence of inflammation.

In our study, exposure to maternal KD resulted in higher brain weight in the offspring at PN21 when compared to the SD. On the other hand, the KD caused significantly lower neuronal density in the PFC and DG at both PN11 and PN21 when compared to the SD group. Sussman observed various region-based neuro-anatomical impacts of the KD on offspring brains, including cerebellar volumetric enlargement with hypothalamic and corpus callosal reduction [20]. The KD seems to have differential effects on different parts of the brain. Although the structural effects of antenatal KD on the developing brain were previously explored in detail, utilizing various imaging techniques [7,20,21], the underlying mechanisms of this region-specific outcome are poorly understood. 

We observed an elevated expression of microglial marker AIF1 in the PC, CA1 and DG regions in the KD group when compared to the SD group at PN11. Our results imply that the KD results in microgliosis and interferes with the normal development of the embryonic brain. In the developing fetus, microglial cells are reported to colonize the human cerebrum between the 4th and 24th gestational weeks [24] and embryonic neurogenesis tightly controlled by microglial cells [25]. The survival and differentiation of neuronal progenitors is highly dependent on proper microglial surveillance. Microglia secrete various neurotrophic factors during development [26,27] and in response to environmental stimuli, displaying different phenotypes with diverse behavior [28,29]. Alterations in the function of fetal microglia are possibly the consequence of external insults; for example, recently, maternal immune activation has been shown to alter fetal microglial motility which later accompanied sustained behavioral differences in late adolescent offspring [30]. A high-fat, high-sugar diet during pregnancy has been found to impair neurogenesis, activate microglia and raise anxiety levels in the offspring [19]. Exposure to the KD leads to the release of ketone bodies into the bloodstream, which they may easily cross via the blood–brain barrier [31] and thus act on the brain. A recent study successfully showed that BHB induces microglial ramification, which promotes anti-inflammatory effects in adult animals [32]. According to another study, pretreatment with the KD attenuated neuroinflammation through the suppression of microglial activation and transformation of microglia from the M1 to M2a phenotype in a rat model of spinal cord injury [33]. Although the anti-inflammatory phenotype is neuroprotective following an insult in adults, the effects of alterations in fetal microglia during the embryonic period are not well studied. Microglia actively seek signals from the external environment and display quick responses which enable for the dynamic shaping of neuronal circuits during development via synapse generation or pruning. Normal embryonic development of the fetal brain requires a delicate balance of microglia, especially in number and function. Disturbing this natural balance in favor of a certain phenotype/function would obviously affect related vital processes such as neurogenesis, neuronal migration, synaptic pruning or the phagocytosis of cellular debris [34]. 

The NLRP3 inflammasome is a multimeric protein complex, and upon activation, it interacts with adaptor protein ASC (apoptosis-associated spec-like protein containing a caspase activating and recruiting domain) which recruits procaspase-1. Procaspase-1 is then converted into mature caspase-1 which cleaves pro-IL-1β and pro-IL-18 into their active forms [35]. We observed that both NLRP3 and IL-1β expression were significantly higher in the KD group when compared to the SD group, both at PN11 and at PN21. Cellular responses following LPS insult under the KD were not studied previously. We observed that a challenge with LPS resulted in more prominent brain injury in the SD group when compared to the KD group. The brain weight of the pups in the SD + LPS group were significantly lower than those in the KD + LPS group at PN11. Additionally, NLRP3 and IL-1β expressions in the DG region were lower in the KD + LPS group when compared to the SD + LPS group at PN21. The KD significantly blunted NLRP3 expression in the presence of LPS insult in the DG region. 

By acting as alternative sources of ATP, the ketone bodies aid mammalian life under conditions of energy deficiency. Starvation, calorie restriction, vigorous activity and the KD all cause an increase in the levels of circulating ketone bodies such as BHB and acetoacetate. Long-term fasting is well known to decrease inflammation [36], and BHB is previously reported to inhibit macrophage NLRP3 inflammasome activation and attenuate caspase-1 activation and IL-1β release in macrophages [36]. According to Kong et al., pretreatment with the KD attenuated neuroinflammation through the suppression of microglial activation, the downregulation of NLRP3 expression and the transformation of the microglia phenotype to the anti-inflammatory phenotype in a rat model of spinal cord injury [33]. In this study, when compared to the rats in the conventional diet group, the rats in the KD group showed better behavioral and electrophysiological recovery following spinal cord injury. In addition, LPS + ATP-induced inflammatory response was suppressed by BHB, and NLRP3 protein levels were lowered in the in vitro experiments done on BV2 cells. Authors claim that BHB could provide a potential therapeutic strategy for spinal cord injury in the future. However, in the present study, unlike adult models, NLRP3 expression was higher in the KD group when compared to the SD group. In the offspring, the DG, CA1, and PFC areas showed increased microglial cells and enhanced NLRP3 expression following exposure to the KD, which operated rather as an insult to the developing brain. Embryonic microglia have multifaceted functions in the developing brain, and molecular mechanisms underlying embryonic microglial behaviors may be completely different from adult microglia [37]. Alternatively, NLRP3 expression was significantly lower in the KD + LPS group when compared to the SD + LPS group in the DG and CA1 regions. This diversity should also be investigated further.

One contributing factor to the region-specific nature of the results may be microglial heterogeneity. Recent studies indicate that the abundance, morpho-molecular characteristics and homeostatic roles of microglia vary in different regions of the brain. Microglial cells exhibit fundamental differences in terms of cell count, cellular and subcellular organization, molecular identity and functional significance [38]. Interactions with neurons or brain progenitor cells as well as the microenvironment would contribute to regional variations in microglia. It is well known that in vitro cultivation of microglia causes significant changes in their morphology, gene signature and activities, indicating their extreme susceptibility to environmental changes. These varied characteristics may cause microglia to react differently to pathogenic insults depending on the location, as well as maybe depending on the time and developmental stage [38]. In our study, regional variations in AIF staining and NLRP3 expression in response to the KD and LPS may result from microglial heterogeneity.

In the present study, we observed similar maternal weight gain patterns among groups during pregnancy; however, maternal weight gain in the KD group was lower than the control group during the lactation period. According to Sussman et al., lactation puts the dams under significant physiological stress in the KD group [20]. They reported that animals in the KD group displayed signs of extreme stress soon after giving birth and died a few days after giving birth because of ketoacidosis, which quickly developed into a lethal metabolic condition. KD pups were consuming milk; however, either the amount or the nutritional value of the maternal KD milk proved insufficient to sustain them. Since the pups in the KD group displayed significant growth retardation, they changed the experimental design and included a fostering process at PN2 to assure the survival of both the KD mothers and pups after parturition. In our study, although we observed lower maternal weight gain during the lactation period, we were able to continue without loss. In line with Sussman et al., offspring in the KD group displayed lower body weight gain on PN2 and PN10; however, the body weights of the offspring were similar in both groups at PN20 in our study. Whether the offspring in the KD group adapted to this situation and experienced catch-up growths, or whether the ones that were strong enough to survive also experienced better catch-up growths, should be investigated further. Kosiek et al. also reported lower body weights in the offspring of the KD group [22]. These discrepancies may also result from variations between the composition of the experimental KD among different studies. In our study, the diet had a higher protein content when compared to that of the Kosiek and Sussman et al. study [7,21,22]. In addition, although our KD group had lower blood glucose levels when compared to the control, we did not observe hypoglycemia in any group. Additionally, in our research, PN11 was the first time point for which data were gathered. Particularly in light of the fact that the KD group at PN21 appeared more prominent than at PN11, there could be at least one component of post-partum mechanisms that may contribute to the transplacental effects of the KD. It is also crucial to distinguish between the antenatal and postnatal mechanisms.

## 5. Conclusions

Results of our study reveal that the KD during pregnancy and lactation negatively affects offspring brains in the mouse model. The effects of the KD exhibited regional variations. On the other hand, in the presence of maternal KD exposure, NLRP3 expression in the offspring brain after LPS injection was lower in the DG and CA1 areas but not in the PFC when compared to the SD group. Further experimental and clinical studies are warranted to elucidate the molecular mechanisms underlying the impact of antenatal KD exposure and regional discrepancies on the developing brain.

## Figures and Tables

**Table 1:** Maternal body weight.

Body Weight (g)	SD (n = 5)		KD (n = 7)		*p*
	$\bar{x}$ ± M	IQR (25–75%)	$\bar{x}$ ± M	IQR (25–75%)	
GD0	22.64 ± 22.6	21.2–24.1	21.71 ± 21	20.6–23	0.43
GD10	25.52 ± 26.4	22.3–28.3	24.89 ± 25	23–26.8	0.75
GD20	36.72 ± 39	32.8–39.5	35.63 ± 36	33.8–38.2	0.53
PP0	26.92 ± 28	24–29.3	24.97 ± 24.2	22.6–27	0.34
PP10	30.16 ± 30.6	28.1–32	26.63 ± 26.8	25.4–29.2	0.04 *
PP20	29.32 ± 30.6	26–32	25.51 ± 25.6	23.4–27	0.07 *

* Values are presented as the means ± medians and 25th–75th quartiles (25–75%).

**Table 2:** Maternal blood β-hydroxybutyrate (BHB) levels.

BHB (mmol/L)	SD		KD		*p*
	$\bar{x}$ ± M	IQR (25–75%)	$\bar{x}$ ± M	IQR (25–75%)	
GD2	0.74 ± 0.8	0.55–0.9	1.24 ± 1.3	1.1–1.3	0.003 *
GD10	0.80 ± 0.8	0.7–0.9	1.70 ± 1.6	1.4–2.1	0.003 *
GD18	0.72 ± 0.7	0.6–0.85	2.80 ± 2.2	2.1–3.3	0.003 *

* Values are presented as the means ± medians and 25th–75th quartiles (25–75%).

**Table 3:** Maternal blood glucose levels.

Glucose (mg/dL)	SD		KD		*p*
	$\bar{x}$ ± M	IQR (25–75%)	$\bar{x}$ ± M	IQR (25–75%)	
GD2	176.0 ± 175	166.5–186	185.4 ± 189	184–192	0.149
GD10	165.0 ± 170	154–173.5	159.6 ± 159	155–169	0.75
GD18	170.8 ± 175	164.5–175	109.6 ± 108	98–115	0.003 *

* Values are presented as mean ± median and 25th–75th quartile (25–75%).

**Table 4:** Maternal total cholesterol and triglyceride levels at GD20.

	SD		KD		*p*	Z
	$\bar{x}$ ± M	IQR (25–75%)	$\bar{x}$ ± M	IQR (25–75%)		
Total cholesterol (mg/dL)	71.80 ± 71.0	57.5–86.5	121.33 ± 118,5	57.5–86.5	0.006 *	−2.739
Trigyceride (mg/dL)	59.80 ± 56.0	53–68.5	109.67 ± 101.50	72–121	0.003 *	−2.191

* Values are presented as the means ± medians and 25th–75th quartiles (25–75%).

**Table 5:** Body weights (g) in the offspring.

Groups	PN2		PN10		PN20	
	$\bar{x}$ ± M	IQR (25–75%)	$\bar{x}$ ± M	IQR (25–75%)	$\bar{x}$ ± M	IQR (25–75%)
SD (1)	1.58 ± 1.60 (n = 19)	1.4–2.0	4.36 ± 4.6 (n = 19)	4.0–5.0	8.44 ± 8.60 (n = 9)	7.5–9.0
KD (2)	1.47 ± 1.60 (n = 25)	1.2–2.0	4.82 ± 5.0 (n = 25)	4.0–5.6	9.56 ± 9.50 (n = 12)	8.6–10.75
SD + LPS (3)	1.69 ± 1.60 (n = 15)	1.4–1.8	3.77 ± 3.6 (n = 15)	2.6–4.2	7.20 ± 8.00 (n = 7)	5.6–8.0
KD + LPS (4)	1.77 ± 1.8 (n = 14)	1.6–2.0	4.94 ± 5.2 (n = 14)	4.4–5.8	10.49 ± 10.60 (n = 7)	9.6–11.2
*p*	0.05		0.000 *		0.008 *	
			(1) > (3), (2) > (3), (4) > (3)		(3) < (4)	

* Values are presented as the means ± medians and 25th–75th quartiles (25–75%).

**Table 6:** Brain weights (g) in the offspring.

Groups	PN11		PN21	
	$\bar{x}$ ± M	IQR (25–75%)	$\bar{x}$ ± M	IQR (25–75%)
SD (1)	0.27 ± 0.27 (n = 10)	0.24–0.31	0.33 ± 0.32 (n = 9)	0.30–0.35
KD (2)	0.28 ± 0.26 (n = 12)	0.21–0.34	0.47 ± 0.49 (n = 12)	0.41–0.51
SD + LPS (3)	0.22 ± 0.22 (n = 8)	0.19–0.25	0.32 ± 0.03 (n = 7)	0.28–0.38
KD + LPS (4)	0.32 ± 0.33 (n = 7)	0.29–0.37	0.32 ± 0.32 (n = 7)	0.31–0.34
*p*	0.021 *		0.003 *	
	(3) < (4)		(1) < (2), (2) > (3), (2) > (4)	

* Values are presented as the means ± medians and 25th–75th quartiles (25–75%).

## Data Availability

Data available on request.

## References

[1] Li M., Francis E., Hinkle S.N., Ajjarapu A.S., Zhang C. (2019). Preconception and Prenatal Nutrition and Neurodevelopmental Disorders: A Systematic Review and Meta-Analysis. Nutrients.

[2] Edlow A.G., Guedj F., Sverdlov D., Pennings J.A., Bianchi D.W. (2019). Significant Effects of Maternal Diet during Pregnancy on the Murine Fetal Brain Transcriptome and Off-spring Behavior. Front. Neurosci..

[3] Prado E.L., Dewey K.G. (2014). Nutrition and brain development in early life. Nutr. Rev..

[4] Mhanna A., Mhanna M., Beran A., Al-Chalabi M., Aladamat N., Mahfooz N. (2022). Modified Atkins Diet versus Ketogenic Diet in Children with Drug-Resistant Epilepsy: A Meta-Analysis of Comparative Studies. Clin. Nutr. ESPEN.

[5] Kishk N.A., Yousof H.Z., Ebraheim A.M., Elkholy T.A.F.A., Soliman S.H., Mohammed R.A., Shamloul R.M. (2021). The effect of ketogenic diet escalation in adolescents and adults with drug-resistant epilepsy: A prospective study. Nutr. Neurosci..

[6] Valenzuela P.L., Castillo-García A., Lucia A., Naclerio F. (2021). Effects of Combining a Ketogenic Diet with Resistance Training on Body Composition, Strength, and Mechanical Power in Trained Individuals: A Narrative Review. Nutrients.

[7] Sussman D., van Eede M., Wong M.D., Adamson S.L., Henkelman M. (2013). Effects of a ketogenic diet during pregnancy on embryonic growth in the mouse. BMC Pregnancy Childbirth.

[8] Maysinger D., Zhang I. (2016). Nutritional and Nanotechnological Modulators of Microglia. Front. Immunol..

[9] Hornedo-Ortega R., Cerezo A.B., De Pablos R.M., Krisa S., Richard T., García-Parrilla M.C., Troncoso A.M. (2018). Phenolic Compounds Characteristic of the Mediterranean Diet in Mitigating Microglia-Mediated Neuroinflammation. Front. Cell Neurosci..

[10] Song L., Pei L., Yao S., Wu Y., Shang Y. (2017). NLRP3 Inflammasome in Neurological Diseases, from Functions to Therapies. Front. Cell Neurosci..

[11] Ghosh S., Castillo E., Frias E.S., Swanson R.A. (2017). Bioenergetic regulation of microglia. Glia.

[12] Shippy D.C., Wilhelm C., Viharkumar P.A., Raife T.J., Ulland T.K. (2020). β-Hydroxybutyrate inhibits inflammasome activation to attenuate Alzheimer’s disease pathology. J. Neuroinflamm..

[13] Percie du Sert N., Ahluwalia A., Alam S., Avey M.T., Baker M., Browne W.J., Clark A., Cuthill I.C., Dirnagl U.l., Emerson M. (2020). Reporting animal research: Explanation and elaboration for the ARRIVE guidelines 2.0. PLoS Biol..

[14] Grochowska K., Przeliorz A. (2022). The Effect of the Ketogenic Diet on the Therapy of Neurodegenerative Diseases and Its Impact on Improving Cognitive Functions. Dement. Geriatr. Cogn. Disord. Extra.

[15] Paxinos G., Watson C. (2006). The Rat Brain in Stereotaxic Coordinates: Hard Cover Edition.

[16] Micili S.C., Engür D., Genc S., Ercan I., Soy S., Baysal B., Kumral A. (2020). Oxygen exposure in early life activates NLRP3 inflammasome in mouse brain. Neurosci. Lett..

[17] Micili S.C., Goker A., Sayin O., Akokay P., Ergur B.U. (2013). The effect of lipoic acid on wound healing in a full thickness uterine injury model in rats. Histochem. J..

[18] Bordeleau M., de Cossío L.F., Chakravarty M.M., Tremblay M. (2021). From Maternal Diet to Neurodevelopmental Disorders: A Story of Neuroinflammation. Front. Cell Neurosci..

[19] Xavier S., Soch A., Younesi S., Malik S., Spencer S.J., Sominsky L. (2021). Maternal diet before and during pregnancy modulates microglial activation and neurogenesis in the post-partum rat brain. Brain Behav. Immun..

[20] Sussman D., Ellegood J., Henkelman M. (2013). A gestational ketogenic diet alters maternal metabolic status as well as offspring physiological growth and brain structure in the neonatal mouse. BMC Pregnancy Childbirth.

[21] Sussman D., Germann J., Henkelman M. (2015). Gestational ketogenic diet programs brain structure and susceptibility to de-pression & anxiety in the adult mouse offspring. Brain Behav..

[22] Kosiek W., Rauk Z., Szulc P., Cichy A., Rugieł M., Chwiej J., Janeczko K., Setkowicz Z. (2022). Ketogenic diet impairs neurological development of neonatal rats and affects biochemical composition of maternal brains: Evidence of functional recovery in pups. Anat. Embryol..

[23] Arqoub A.M.S., Flynn K.G., Martinez L.A. (2020). Gestational exposure to a ketogenic diet increases sociability in CD-1 mice. Behav. Neurosci..

[24] Menassa D.A., Gomez-Nicola D. (2018). Microglial Dynamics during Human Brain Development. Front. Immunol..

[25] Tong C.K., Vidyadaran S. (2016). Role of microglia in embryonic neurogenesis. ExBiol. Med..

[26] Sominsky L., De Luca S., Spencer S.J. (2018). Microglia: Key players in neurodevelopment and neuronal plasticity. Int. J. Biochem. Cell Biol..

[27] Wright-Jin E.C., Gutmann D.H. (2019). Microglia as Dynamic Cellular Mediators of Brain Function. Trends Mol. Med..

[28] Boche D., Perry V.H., Nicoll J.A.R. (2013). Review: Activation patterns of microglia and their identification in the human brain. Neuropathol. Appl. Neurobiol..

[29] Eren E., Tufekci K.U., Isci K.B., Tastan B., Genc K., Genc S. (2018). Sulforaphane Inhibits Lipopolysaccharide-Induced Inflammation, Cytotoxicity, Oxidative Stress, and miR-155 Expression and Switches to Mox Phenotype through Activating Extracellular Signal-Regulated Kinase 1/2-Nuclear Factor Erythroid 2-Related Factor 2/Antioxidant Response Element Pathway in Murine Microglial Cells. Front. Immunol..

[30] Ozaki K., Kato D., Ikegami A., Hashimoto A., Sugio S., Guo Z., Shibushita M., Tatematsu T., Haruwaka K., Moorhouse A.J. (2020). Maternal immune activation induces sustained changes in fetal microglia motility. Sci. Rep..

[31] Pierre K., Pellerin L. (2005). Monocarboxylate transporters in the central nervous system: Distribution, regulation and function. J. Neurochem..

[32] Huang C., Wang P., Xu X., Zhang Y., Gong Y., Hu W., Gao M., Wu Y., Ling Y., Zhao X. (2017). The ketone body metabolite β-hydroxybutyrate induces an antidepression-associated ramification of microglia via HDACs inhibition-triggered Akt-small RhoGTPase activation. Glia.

[33] Kong G., Liu J., Li R., Lin J., Huang Z., Yang Z., Wu X., Huang Z., Zhu Q., Wu X. (2020). Ketone Metabolite β-Hydroxybutyrate Ameliorates Inflammation After Spinal Cord Injury by Inhibiting the NLRP3 Inflammasome. Neurochem. Res..

[34] Arcuri C., Mecca C., Bianchi R., Giambanco I., Donato R. (2017). The Pathophysiological Role of Microglia in Dynamic Surveillance, Phagocytosis and Structural Remodeling of the Developing CNS. Front. Mol. Neurosci..

[35] Hanslik K.L., Ulland T. (2020). The Role of Microglia and the Nlrp3 Inflammasome in Alzheimer’s Disease. Front. Neurol..

[36] Youm Y.-H., Nguyen K.Y., Grant R.W., Goldberg E.L., Bodogai M., Kim D., D’Agostino D., Planavsky N., Lupfer C., Kanneganti T.-D. (2015). The ketone metabolite β-hydroxybutyrate blocks NLRP3 inflammasome–mediated inflammatory disease. Nat. Med..

[37] Hattori Y. (2021). The behavior and functions of embryonic microglia. Anat. Sci. Int..

[38] Tan Y.-L., Yuan Y., Tian L. (2019). Microglial regional heterogeneity and its role in the brain. Mol. Psychiatry.

